# Patient-derived colorectal microtumors predict response to anti-PD-1 therapy

**DOI:** 10.3389/fimmu.2025.1640500

**Published:** 2025-08-12

**Authors:** Duy T. Nguyen, Matthew A. Schaller, Krista P. Terracina, Xia Xu, Diego I. Pedro, Alfonso Pepe, Juan M. Urueña, Zadia Dupee, Nickolas Diodati, Ryan A. Smolchek, Jack E. Famiglietti, Nhi Tran Yen Nguyen, Gerik W. Tushoski-Alemán, Kuoyuan Cheng, Lan Chen, Doug Linn, Vania Vidimar, Aquila Fatima, Soon Woo Kwon, Dongyu Sun, Hongmin Chen, Haiyan Xu, Brian Long, Lily Y. Moy, Bonnie J. Howell, George H. Addona, W. Gregory Sawyer

**Affiliations:** 1 Department of Mechanical and Aerospace Engineering, University of Florida, Gainesville, FL, United States; 2 Division of Pulmonary, Critical Care, and Sleep Medicine, University of Florida, Gainesville, FL, United States; 3 Department of Surgery, College of Medicine, University of Florida, Gainesville, FL, United States; 4 Department of Quantitative Biosciences, Merck & Co., Inc., Rahway, NJ, United States; 5 MRL, Discovery, Preclinical and Translational Medicine, Merck & Co., Inc., Rahway, NJ, United States

**Keywords:** colorectal cancer, CRC, microsatellite instability, immune checkpoint inhibitor, immunotherapy, anti-PD-1, patient-derived explants, *ex vivo*

## Abstract

Immune checkpoint inhibitors have made remarkable impacts in treating various cancers, including colorectal cancer (CRC). However, CRC still remains a leading cause of cancer-related deaths. While microsatellite instability (MSI) CRC has shown positive responses to anti-PD-1 therapy, this subgroup represents a minority of all CRC patients. Extensive research has focused on identifying predictive biomarkers to understand treatment response in CRC. Interestingly, a growing number of clinical cases have reported favorable outcomes from a subtype of supposedly non-responder microsatellite stable (MSS) CRC, characterized by DNA polymerase ϵ (POLE) proofreading domain mutations with high tumor mutational burden (TMB). This subtype has shown a notable response, either partial or complete, to pembrolizumab as salvage treatment, often following significant disease progression. To improve efficiency, cost-effectiveness, and clinical outcomes, there is an essential need for a testing platform capable of promptly identifying evidence of anti-PD-1 response to inform treatment strategies. Here, we established a novel 3D *ex vivo* immunotherapy model using patient-derived tumor microexplants (or microtumors <1 mm) co-cultured with autologous peripheral blood mononuclear cells (PBMCs) from treatment-naïve CRC patients. We demonstrate that long-term *ex vivo* treatment with pembrolizumab induced a heterogeneous but appreciable interferon-gamma (IFN-γ) secretion, accompanied by infiltrating PBMCs. Intriguingly, a case study involving an MSS CRC phenotype harboring *POLE* mutation and associated ultrahigh TMB demonstrated a response to PD-1 blockade, potentially from the intratumoral immune cell population. Ultimately, this novel model could serve as a valuable tool in complementing clinical diagnostics and guiding personalized treatment plans for CRC patients, particularly those with specific phenotypes and mutational profiles.

## 1 Introduction

The initial success of programmed cell death 1 (PD-1) blockade in advanced colorectal cancer (CRC) patients was primarily demonstrated in patients with DNA mismatch repair deficiency, leading to FDA approval in 2017 for the treatment of unresectable or metastatic microsatellite instability-high (MSI-H) solid tumors in both adult and pediatric patients (1, 2). This marked the first time that the FDA approved a cancer treatment based on a biomarker instead of the primary site of origin. Despite these advancements, CRC remains one of the deadliest cancers worldwide, and a substantial number of patients are still ineligible for or do not benefit from anti-PD-1 therapy.

The significantly elevated tumor mutation burden (TMB) (≥10 mutations per megabase), observed in MSI CRC and across various cancer types is linked to a favorable response to immune checkpoint inhibitor therapy (3–5). This contributes to an enhanced prognosis for MSI CRC patients compared to those with microsatellite-stable (MSS) phenotypes (3, 6, 7). However, response to anti-PD-1 remains variable, even among MSI CRC patients (8). In a clinical study conducted in 2017, the objective response rate for anti-PD-1 treatment in advanced MSI cancers was found to be 53%, with 21% of patients achieving complete remission (2). Furthermore, the presence of intratumor and interpatient heterogeneity (9, 10), contributes to therapeutic resistance, even within well-defined responsive phenotypes (8). This underscores the important role of personalized medicine in optimizing treatment approaches (11).

MSS CRC is generally considered a non-responder phenotype to anti-PD-1 and represents the majority of CRC patients. Extensive research has been focused on identifying predictive biomarkers that can help determine the efficacy of checkpoint inhibitors, either as monotherapy or in combination with other therapeutic modalities for the treatment of MSS CRC. Recent studies have highlighted the significance of germline and somatic mutations in the DNA polymerase ϵ (*POLE*), particularly within the exonuclease (proofreading) domain codons 268-471, in contributing to high TMB. These mutations may serve as potential indicators of response to immune checkpoint inhibitor therapy (12). Mutations within the exonuclease domain of the *POLE* gene disrupt its proofreading function, resulting in subsequent hypermutation (13), which positively correlates to neoantigen burden and response to immunotherapy (14). Furthermore, clinical case studies have reported favorable responses to PD-1 blockade in MSS CRC patients carrying *POLE* mutations and exhibiting high TMB (**Table 1**) (15–18). Since these patients were diagnosed with MSS CRC phenotype, immunotherapy was an unconventional choice. Anti-PD-1 was only used as a salvage treatment after patients underwent multiple cycles of standard chemotherapy without improvement of disease progression, which generally causes extreme fatigue. Despite the promising outcomes from these cases, somatic mutation of the *POLE* exonuclease domain is rare in CRC (< 3%) and not all *POLE* mutations are associated with response to immune checkpoint therapy (19). In fact, the pathogenicity of the majority of *POLE* mutations, whether inside or outside the exonuclease domain, remains unknown (12). This highlights the necessity for a model to promptly detect evidence of response to anti-PD-1 therapy, especially in CRC patients exhibiting *POLE*-driven hypermutation, who may benefit from immunotherapy.

**Table 1:** Clinical response to ICI treatment in MSS colorectal cancers with *POLE* mutations.

Ref.	Age	Sex	Race	MMR status	Prior to ICI treatment	POLE	TMB/Mb	ICI treatment	Response (months)
(15)	34	M	Asian	MSS	adjuvant chemo	p.P286R	--	pembrolizumab	stable disease (>7)
(16)	81	M	Hispanic	KRAS-MSS	adjuvant chemo	p.V411L	122	pembrolizumab	partial response (>12)
(17)	44	M	--	KRAS-MSS	neoadjuvant radiation	p.V411L	200	pembrolizumab	complete response (> 28)
(18)	29	M	Asian	MSS	adjuvant chemo	p.F367S	103	pembrolizumab	complete response (>49)

Animal models have played a crucial role in advancing oncology research; however, relying solely on *in vivo* models has limitations, including genetic variations, resource-intensive requirements, time constraints, and differences in immune system responses. Complementary to *in vivo* studies, advanced *ex vivo* models have leveraged cutting-edge biotechnological techniques, providing a platform for a more profound mechanistic understanding of cancer development and progression. Integrating both *in vivo* and *ex vivo* approaches allows researchers to gain a comprehensive understanding of cancer biology and therapeutic responses.

Amongst 3D cell models, patient-derived explant is arguably the most representative model of human disease, providing a personalized and individual-specific representation. The *ex vivo* model inherently preserves the intact tissue extracellular matrix (ECM) along with a heterogeneous assembly of cellular components exhibiting diverse phenotypes and genotypes, each with distinct, context-dependent functions (20, 21). This intricate composition closely mirrors the original tumors (21–23). Despite the challenges in maintaining tissue explants *ex vivo*, recent advancements in biotechnology (22, 24–27), have revitalized this model, positioning it as a valuable preclinical tool for both basic and translational research. In previous work, we demonstrated the successful 3D long-term culture of mouse colorectal microexplants (24). The use of perfusion media, along with the removal of metabolic waste, enabled the preservation of cell viability, tissue architecture, and peristaltic-like activity in mouse gut microexplants (24). In another study, we characterized T cell locomotion within our 3D bioconjugated liquid-like solid microgel platform, designed to mimic the physical barrier of the solid tumor microenvironment (28). This enables a more comprehensive understanding of cancer-immune interactions (28).

**Figure 1:**
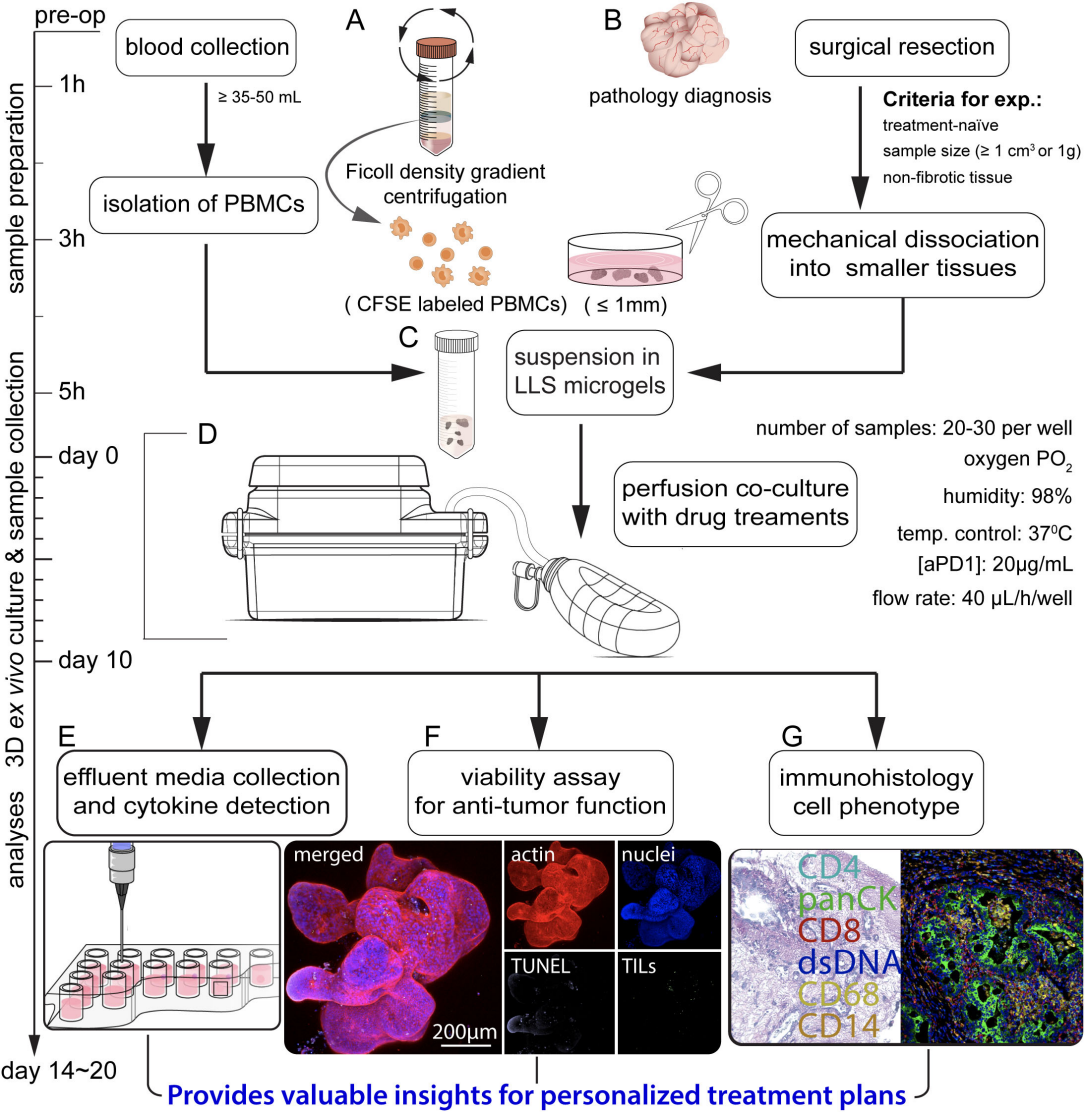
Schematic illustrating the sequential steps in the co-culture of patient-derived microtumors and autologous PBMCs for anti-PD-1 (aPD1) treatment. **(A)** Isolation of PBMCs from whole blood through Ficoll density gradient centrifugation, prelabeling with CFSE. **(B)** Surgical removal and collection of tumor specimens for pathology diagnoses, followed by mechanical dissection into tumor microexplants (namely microtumors; approximately ≤ 1 mm). **(C)** Uniform suspension of PBMCs and microtumors in LLS microgels. **(D)** Co-culture in a perfusion platform under varied treatment conditions. Assessment of immune response involves **(E)** analyzing effluent media for cytokine production, **(F)** evaluating immune infiltration and anti-tumor function, and **(G)** performing immunohistology.

**Figure 2:**
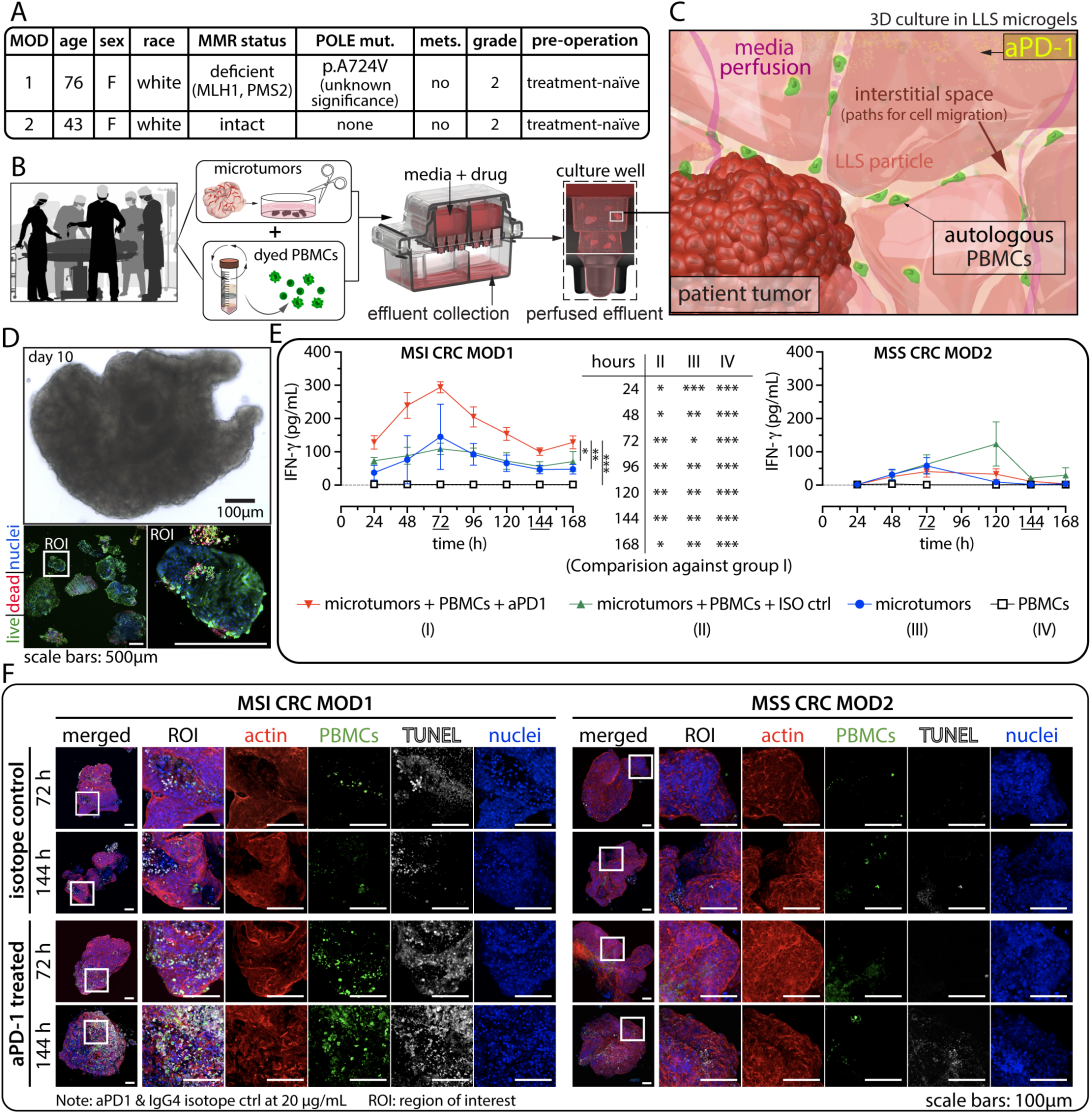
Evaluation of anti-PD-1 (aPD1) in patient-derived *ex vivo* models. **(A)** Patient-specific MMR status and other relevant information. **(B)** Schematic representation of the experimental workflow from blood draw and surgical resection to *ex vivo* co-culture of patient-derived microtumors and autologous PBMCs. **(C)** Illustration depicting the dynamic interaction between microtumors and immune cells within the 3D environment. **(D)** Typical tissue morphology (bright field) and immunofluorescence (IF) viability data using Calcein AM (live, green) and BOBO-3 Iodide (dead, red) after long-term *ex vivo* culture. **(E)** Detection of interferon-gamma (IFNγ) secretion from perfused media collected every 24 hours for 7 days in the 3D tumor-immune cells co-culture for MSI (left) and MSS (right) CRC cases. Statistical significance was shown for MOD1 only. There is no statistical significance for MOD2. Statistical analysis was performed using a two-way ANOVA followed by Tukey’s HSD *post-hoc* test for IFNγ level between the treatment groups at each time point. (technical replicates n=3 unless indicated otherwise, *p<0.05, **p<0.01, ***p<0.001). **(F)** TUNEL assay was employed to detect apoptotic cells, where the presence of CFSE+ immune infiltration correlates with regions of cell death. ROI: region of interest.

**Figure 3:**
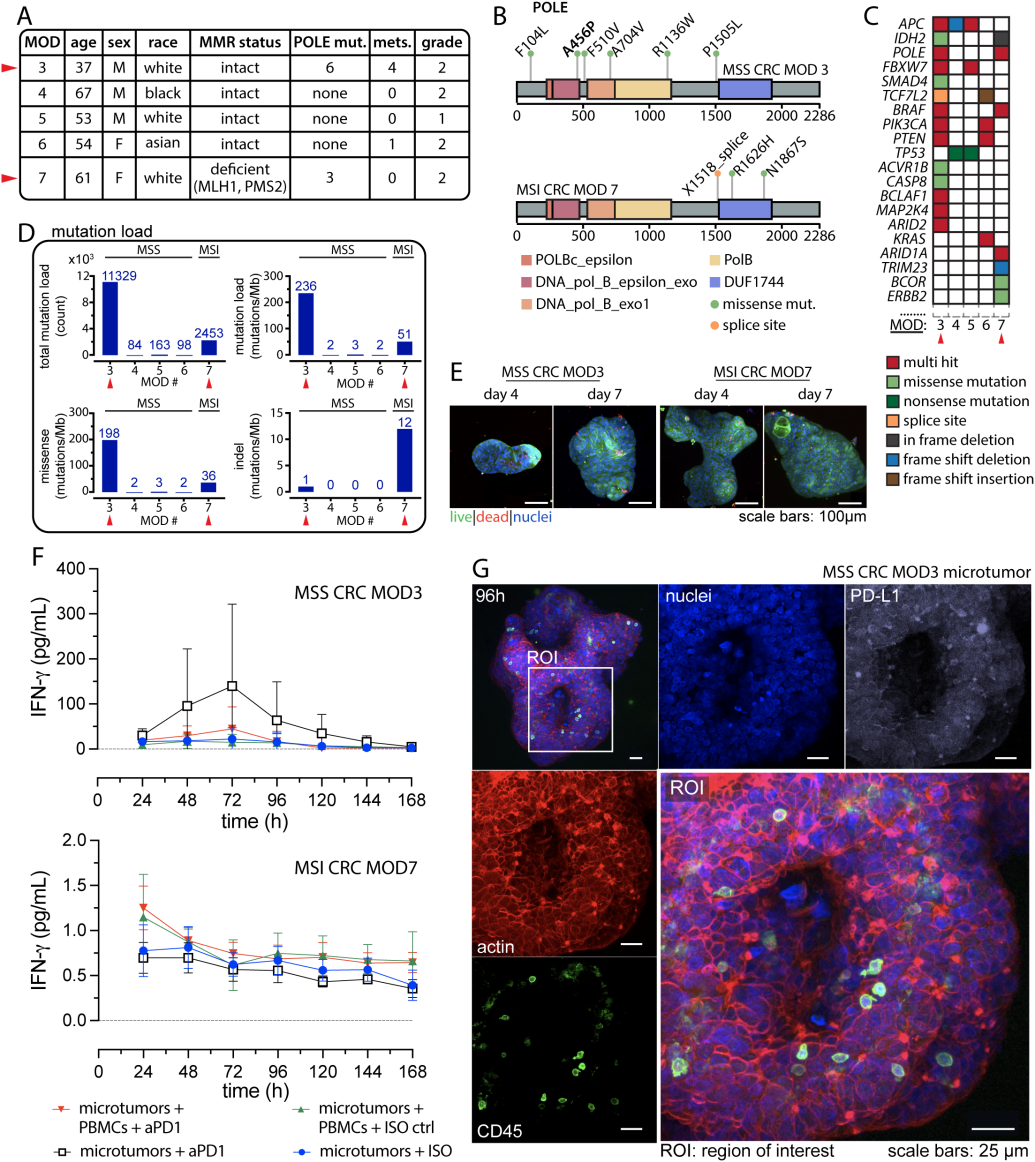
*POLE* mutations potentiate anti-tumor response to anti-PD-1 Treatment. **(A)** Patient information comparing case studies of CRC harboring *POLE* mutation with other MSS CRC cases. **(B)** Distribution of the *POLE* mutations within the *POLE* full-length sequence for MSS CRC MOD3 (top) and MSI CRC MOD7 (bottom). **(C)** Somatic mutation profiles in known cancer genes of CRC for the presented cases. **(D)** Mutation load reveals hypermutation status in MSS CRC with *POLE* mutation compared to other MSS and MSI phenotypes. **(E)** Typical IF viability data using Calcein AM (live, green) and BOBO-3 Iodide (dead, red) on day 4 and day 7 for the two case studies. **(F)** Detection of interferon-gamma (IFNγ) from effluent media collected every 24 hours for 7 days. Technical replicates, n=3 for all conditions. **(G)** Confocal images displaying resident immune cells in microtumors. An ROI highlights the detection of CD45+ intratumoral immune cell populations. Separate confocal channels show distinct fluorescent markers for actin (red), immune cells (green: CD45), nuclei (blue), and PD-L1 (white).

Here, we developed an *ex vivo* model of patient-derived tumor microexplants, namely microtumors co-cultured with autologous peripheral blood mononuclear cells (PBMCs) to screen for evidence of early response to anti-PD-1 treatment (**Figure 1**). The CRC microtumors cultured in our custom-built perfusion platform (24), maintained cell viability and tissue heterogeneity with the presence of endogenous immune populations. Perfusion of pembrolizumab (anti-PD-1) through the co-culture for 7 days elicited IFNγ production and evidence of immune infiltration in responder cases (**Figures 2**, **3**). Notably, we conducted a case study *ex vivo* involving MSS metastatic CRC harboring the *POLE* A456P exonuclease mutation, along with five additional mutations of unknown significance on the *POLE* gene (**Figure 3**). This case exhibited extremely high TMB through whole-exome sequencing (WES). Treatment with anti-PD-1 resulted in a response with IFNγ production. Interestingly, a similar study on MSI CRC with three *POLE* mutations of unknown significance did not elicit an appreciable response. Our *ex vivo* model suggests that CRC patients with *POLE* mutations and high TMB may benefit from immune checkpoint therapy. Our results demonstrated the development of a novel patient-based *ex vivo* model for immune checkpoint inhibitor therapeutic drug screens, aiding the development of personalized treatment plans.

## 2 Materials and methods

### 2.1 Informed consent and ethical approval

This study was conducted in compliance with ethical standards and approved by the University of Florida Institutional Review Board (IRB) under protocol IRB202001363. All subjects were informed on the purpose of the study and gave written informed consent before participation. Informed consent was obtained from all participants or their legal guardians prior to the collection of any tissue sample. The study was carried out in accordance with the protocol and relevant guidelines and regulations, and the experimental protocol was approved by the University of Florida IRB (IRB202001363). No animal models were used during the conduct of this study.

### 2.2 Generation of tissue microexplants

The procedure of microexplant generation was previously reported (24). In brief, the surgically resected specimens were immediately submerged in ice-cold, calcium and magnesium-free PBS (1X) containing 10% FBS and transported to the laboratory within two hours of the surgery. External membranes and observable adipose tissues were removed using sterile forceps and scissors. The samples were washed three times with ice-cold PBS (1X). Holding on to one end of the sample with forceps, the sample was cut into smaller pieces using a surgical scalpel. The pieces were further cut with surgical scissors to sizes no larger than 1 mm. Without enzyme digestion, these microexplants were then transferred to a 15-mL tube containing approximately 10mL of ice-cold PBS and further chilled on ice for at least 5 minutes. The supernatant was discarded once a relatively clear collection of microexplants had settled at the bottom. This procedure was repeated three times to remove single cells and decellularized extracellular matrix (ECM) fragments. The collected microexplants were placed in 3D perfusion culture using pre-wetted wide-bore micropipette tips.

### 2.3 Peripheral blood mononuclear cells collection

Peripheral blood mononuclear cells (PBMCs) were isolated from the patient peripheral blood (~ 30–50 mL) using a Ficoll gradient (Ficoll-Paque Plus; Amersham Biosciences). The cells were labeled with carboxyfluorescein succinimidyl ester - CFSE (Cell Division Tracker Kit, 423801; BioLegend) and counted with a hemocytometer prior to the co-culture experiment.

### 2.4 Microexplants in perfusion culture

Perfusion culture was conducted as previously described (24). In brief, the microtumor samples were suspended in media-equilibrated liquid-like solid (LLS) microgels. A small sample of the microtumor-LLS mixture was placed on an inverted light microscope to count the microtumors per unit volume. Each well of a perfusion plate was loaded with 200µL of LLS containing PBMCs uniformly suspended at 10^6^ cells/mL and approximately 30–40 CRC microtumors (**Figures 1A–D**). After loading, the plate was centrifuged at 100xg for 5 minutes at room temperature with a moderate acceleration and deceleration setting (Thermo Fisher Scientific, Waltham, MA, USA, Sorvall ST 40R). Culture media was carefully added to each quadrant of the plate to avoid disturbing the centrifuged microgels and samples. A 100cc Jackson-Pratt drain (Care Express, Cary, IL, USA, SU130-1305) was connected to establish a pressure gradient and initiate media perfusion as previously described (24). The culture conditions were maintained at 37°C with 5% CO_2_. The perfusion plates were maintained every 24 hours by adding pre-warmed media to the media reservoirs and collecting perfused media from the waste collection compartments.

### 2.5 Experimental conditions

Patient-derived microtumors and autologous PBMC were co-cultured long-term in the presence of pembrolizumab (anti-PD-1). We established three (3) control conditions: co-culture of tumor microexplants and CFSE labelled PBMCs, incorporating a human IgG4 isotype control (ISO ctrl); microtumors or PBMC alone with ISO ctrl or with anti-PD-1. Experimental conditions are clearly defined in the Results and the figures. The analysis was centered on differentiating evidence of immune response from MSI CRC as compared to MSS CRC. The IFNγ production between the responding versus non-responding cases was measured (**Figures 1E–G**). Note: pembrolizumab (anti-PD-1) was kindly provided by Merck & Co., Inc., Rahway, NJ, USA. The concentration of both pembrolizumab and the isotype control (IgG4) was 20 µg/mL for all experiments. Clinically, pembrolizumab achieves steady-state trough serum concentrations of approximately 50 µg/mL in patients receiving the standard 300 mg every two weeks (Q2W) dosing regimen. In *ex vivo* co-culture models, the absence of clearance mechanisms and the defined volume of culture medium enable consistent antibody exposure to target cells. A concentration of 20 µg/mL offers a conservative and effective dose that falls within the biologically active range and has been widely used in previous preclinical studies to block PD-1 signaling and elicit T-cell activation (29, 30).

### 2.6 Immunofluorescence assay

The immunofluorescence (IF) staining protocol was as previously completed in (24, 25). CRC microtumor samples were fixed in 4.0% formaldehyde in 1X PBS overnight at 4°C, washed twice, and incubated in PBS (1X) for 1 hour at room temperature. The samples were then permeabilized in 0.5% Triton X-100 (Sigma-Aldrich, St. Louis, MO, USA, X100-100ML) for 2 hours, washed twice, and blocked with 3% bovine serum albumin in PBS for 3 hours at room temperature. After blocking, samples were washed 3 times with PBS and incubated overnight with conjugated antibodies at 4°C. The antibodies used in this assay included E-cadherin (BD Biosciences, San Jose, CA, USA, 560062), Alexa Fluor^®^ 488 Anti-CD45 antibody (Abcam, Cambridge, UK, ab197730), and Alexa Fluor™ 568 Phalloidin (Invitrogen, Waltham, MA, USA, A12380). After overnight incubation with the antibodies, the samples were washed 3 times with PBS and counterstained with Hoechst 33342 (Invitrogen, Waltham, MA, USA, H3570) for 15 minutes before imaging.

For live-cell viability assay, a live/dead kit (Thermo Fisher, Waltham, MA, USA, R37601) containing Calcein AM and BOBO-3 iodide was used, following the manufacturer’s protocol. Briefly, Calcein AM was gently mixed with BOBO-3 iodide. The solution was added to an equal volume of media-containing samples and incubated at 37°C with 5% CO_2_ for 30 minutes. Hoechst 33342 (Invitrogen, Waltham, MA, USA, H3570) was added to visualize cell nuclei. Subsequently, the samples underwent at least two washes with PBS (1X) before imaging. Samples were submerged in pre-warmed culture media and placed in a custom-built stage incubator for live-cell imaging.

### 2.7 TUNEL assay

Terminal deoxynucleotidyl transferase (TdT) dUTP Nick-End Labeling (TUNEL) assay (Click-iT Plus TUNEL assay, Invitrogen, Waltham, MA, USA, C10619) was applied to detect apoptosis in cancer cells and correlated with infiltrating CFSE labeled PBMCs. The assay was conducted following manufacturer protocol. All CRC microtumors were then imaged using a Nikon A1R HD25 confocal microscope equipped with a high-definition Galvano scanner.

### 2.8 Immunohistochemistry

Formalin-fixed paraffin-embedded (FFPE) tissues were sectioned at 5µm thickness and stained on the Leica Bond autostainer using optimized conditions for CD8 (CST#85336), CD4 (CST#48274), PD-1 (CST#86163), or PD-L1 (Abcam, Cambridge, UK, ab228415) antibodies. Serial sections were subjected to hematoxylin and eosin (H&E) staining.

### 2.9 Multiplexed ion beam imaging and analyses

FFPE tissues were sectioned at 5µm thickness, deparaffinized with xylene, and rehydrated with successive washes in reagent alcohol and water. Antigen retrieval was performed using Tris/EDTA pH9 solution (Dako) prior to blocking with 5% donkey serum. Slides were stained overnight at 4°C using a 25-plex cocktail of antibodies (dsDNA, beta-tubulin, CD298, CD4, CD11C, FOXP3, PD-1, PD-L1, GZMB, CD56, CD31, Ki67, CD14, CD68, CD8, CD3, CD45RO, VIM, alpha-SMA, pan-cytokeratin [pan-CK], CD20, PDPN, HLA-DR, HLA-ABC, and CD45). Following washings with TBS-T, slides were fixed for 5 minutes in 2% glutaraldehyde (Electron Microscopy Sciences), dehydrated with decreasing dilutions of ethanol, and dried prior to acquisition on MIBIscope. Ten (10) 800x800µm regions of interest (ROIs) per sample were selected based on the presence of immune infiltrates in serial H&E stains. The spatial locations of ROIs are clearly defined in the figures.

Following image quality control (QC), ROI regions were quantified as tumor based on pan-CK positivity or stroma based on VIM or alpha-SMA positivity. Watershed segmentation using a combination of nuclear, cytoplasmic, and membrane markers was used for immune population quantification. Cell classification was performed using a machine learning framework with pathologist-defined intensity thresholds for immune lineage markers. Marker intensities were quantified as ion counts per cell and cell densities were quantified as cells per mm^2^. The nearest neighbor analysis quantified the average distance from tumor cells to immune lineages. Statistical analyses were performed with GraphPad Prism8 using a one-way ANOVA with Tukey’s multiple comparison test. A cell density heat map was generated by normalizing population density with a z-score computation. Each column represents a single ROI, and each row reflects a different cell population. Red indicates higher values, and blue indicates lower values, as shown in the color scale.

### 2.10 IFNγ detection

A multi-parameter bead-based ELISA MSD IFNγ Small Spot assay (Meso Scale Diagnostics, MD, Cat#K151TTK-2) was employed to measure the IFNγ level from the daily perfused effluent media from the individual well of the perfusion platform. The assay was conducted following manufacturer protocol.

### 2.11 Somatic mutation

Whole exome sequencing (WES) reads were aligned to human reference genome GRCh37 with Sentieon v201808.06 (31) including post-processing steps (deduplication, indel realignment and base quality recalibration) to generate the bam files for downstream analysis. Somatic mutations in the tumor samples were called with GATK v4.1.4.0 Mutect 2 (32) using the matched tumor-infiltrated lymphocyte (TIL) samples as paired-normal controls. A panel of 522 normal samples was also used. Mutations with total read depth < 15 or mutant allele read depth < 4 were excluded, and the resulting mutations were further filtered with dbSNP b151 (33) and COSMIC v90 (34) databases. Specifically, mutations listed in dbSNP while not present in COSMIC were excluded. VEP v98 (35) was used to annotate the mutations. All protein-altering mutations (including splicing site mutations) were used for calculating mutation load. The length of regions used to calculate the normalized mutation count per megabase value was obtained by intersecting the WES bait regions and refGene exon regions padded with +/- 2 bp for splicing sites. The R package maftools (36) was used to generate the plots of mutations on the POLE protein.

### 2.12 Statistical analysis

**Figure 4:**
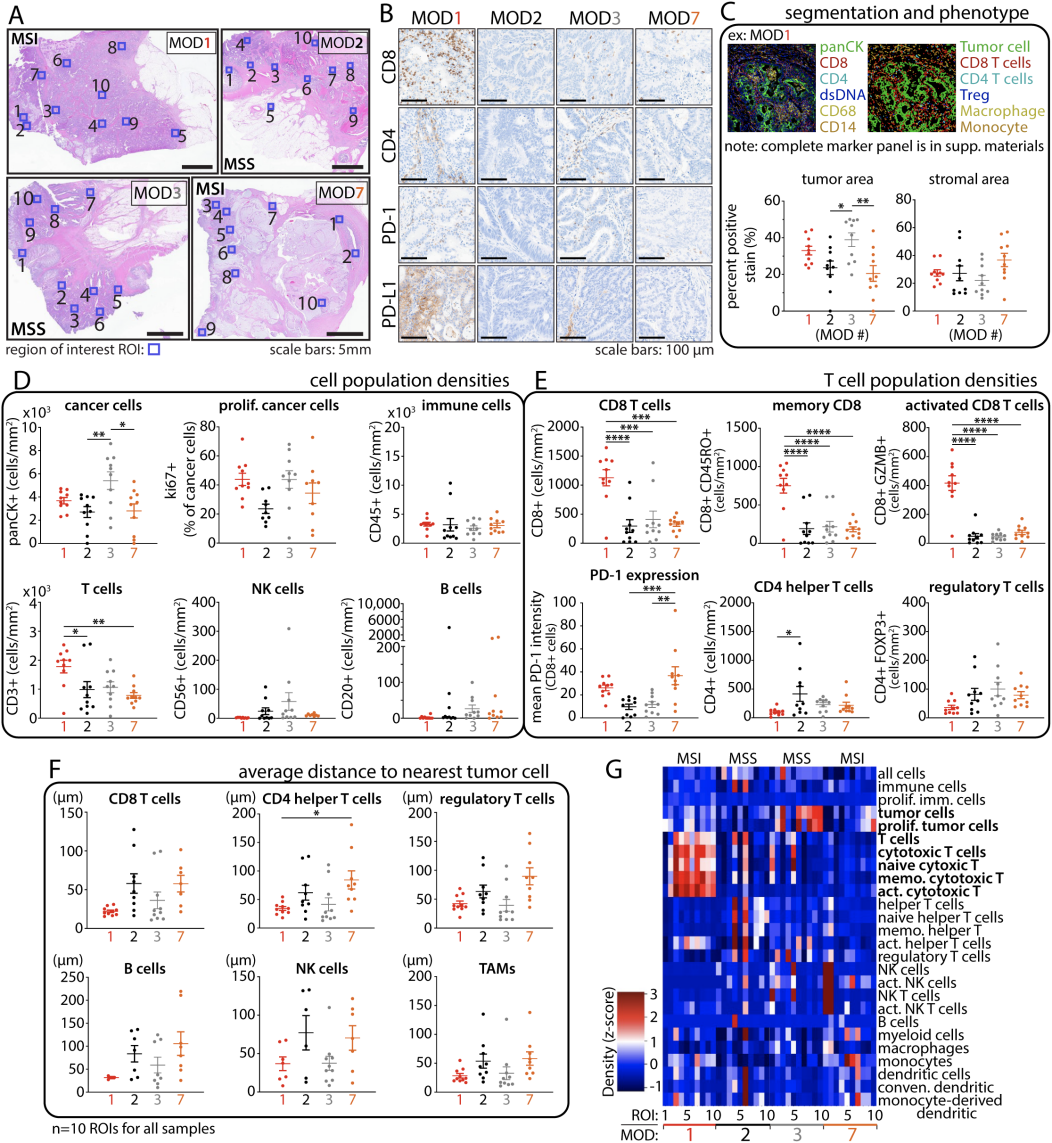
Multiplex immune cell profiling reveals heterogenous intratumoral immune populations in CRC tumors. **(A)** Histology images depict individual tumors with regions of interest (ROIs, blue box, n=10) selected for immune cell profiling. **(B)** IHC staining of TILs and immune checkpoint expression. **(C)** Representative example of cell segmentation and immune phenotyping. Tumor and stromal areas exhibit strong agreement with histology images from A. **(D)** Cell population densities (cells per mm^2^) show heterogeneous distribution among patients, even in those with similar CRC phenotypes. **(E)** T-cell population densities. **(F)** Proximity analysis reveals closer T cell distance to tumor cells in MOD1 (MSI) and MOD3 (MSS *POLE*) compared to other cases. **(G)** Cell density heat map reveals a high proportion of T cells in MOD1 compared to the rest of the samples. Statistical analysis was performed using One-Way ANOVA followed by Tukey multiple comparison test; *p < 0.05, **p < 0.01, ***p < 0.001, and ****p < 0.0001.

GraphPad Prism software was used for statistical analysis. For **Figure 2**, we used a two-way ANOVA followed by Tukey’s HSD posthoc test for IFNγ level between the treatment groups at each time point. For **Figure 4**, we used one-way ANOVA with multiple comparison tests for multiple-group comparisons. Statistical analysis was also indicated in the figure captions.

## 3 Results

### 3.1 Co-culture of patient-derived CRC microtumors and autologous PBMCs

The CRC cases were selected for the *ex vivo* model based on the following criteria: being treatment-naïve, having a sufficient blood draw (at least 35–50 mL) for PBMC isolation, and having a sufficient sample size (≥ 1 cm^3^ or 1g) without observable fibrotic tissue (**Figures 1A, B**). Tissue specimens were collected from properly consented colorectal cancer patients enrolled in our research program, Microtissues for Oncology and Drug Discovery (MODEL or MOD) (**Figure 2A**, **Supplementary Table S1**).

After surgical resection, specimens were promptly collected and delivered to the pathology department for analysis. Subsequently, a portion of the tissue was forwarded to our laboratory for experiment. The specimens were mechanically dissociated into smaller tissue fragments (≤ 1 mm) and co-cultured with CFSE-labeled PBMCs (10^6^ cells/mL) as shown in the workflow in **Figures 1A–D** and **2B**. In previous work, we demonstrated the capacity of T cells to navigate through the interstitial space of a Liquid-Like Solid (LLS) material, then infiltrate, and effectively eliminate target tumors in a 3D static co-culture (28). Building upon these previous findings (28), our objective was to construct an *ex vivo* model involving the co-culture of patient-derived tumors and immune cells to assess the anti-tumor response to Immune Checkpoint Inhibitor (ICI) therapy. We also employed a custom-built 3D platform to support the *ex vivo* co-culture of cells and tissues through the mechanism of media perfusion transport (24). In the culture chamber, the LLS provides a stable 3D support structure for immune cells and tumor microexplants, referred to as microtumors interchangeably in this study (28). The interstitial space between microgel particles facilitates cell motility and allows the perfusion of media (24, 28), (**Figure 2C**). The platform ensures a constant supply of culture media and the removal of waste metabolites through a continuous flow (24).

We observed a heterogeneous microtumor morphology with an intact tumor boundary, confirming the presence of viable cell populations and indicating signs of proliferation and self-organization over time, as typically shown in **Figures 1F**, **2D**. To evaluate immune cell infiltration, the co-cultured samples were collected and thoroughly washed with PBS (1X) to remove surrounding non-infiltrating PBMC cells. Consequently, the detectable CFSE+ PBMC population in the microtumors exclusively represents infiltrated immune cells after long-term co-culture (**Supplementary Figure S1**). In summary, we demonstrated that microtumors and autologous PBMCs remained viable over an extended co-culture in our perfusion platform, making them suitable for drug screening.

### 3.2 *Ex vivo* response to pembrolizumab: MSI *vs* MSS CRC

We co-cultured patient-derived CRC microtumors with autologous CFSE-labeled PBMCs and treated them with pembrolizumab (defined as microtumors + PBMCs + anti-PD-1) at 20 µg/mL under perfusion. We established three control conditions to assess anti-tumor response: (1) co-culture of tumor microexplants and CFSE labelled PBMCs, incorporating a human IgG4 isotype control (defined as microtumors+ PBMCs+ ISO ctrl) at 20 µg/mL. This critical control ensured that any observed anti-tumor response would be specific to the PD-1 blockade. In addition, (2) microtumors alone and (3) PBMCs alone were used to evaluate the baseline anti-tumor activities from pre-existing Tumor-Infiltrating Lymphocyte (TILs) populations and the patient’s immune system respectively.

The levels of IFNγ in the daily perfused media were analyzed. IFNγ serves as a key indicator of immune-mediated anti-tumor function resulting from anti-PD-1 treatment. A significant elevation in IFNγ levels was observed in CRC microtumors-PBMC co-culture following anti-PD-1 treatment in MSI CRC MOD1 (**Figure 2E**). The IFNγ level remained consistently higher than in all three control conditions throughout the study, reaching its peak at 72 hours. This suggests a potential recovery period for these biological samples post-resection, tissue process, and *ex vivo* culture. Furthermore, the IFNγ level in MSS CRC MOD2 coculture, which was expected to be a non-responder to anti-PD-1 therapy, remained consistently low across all treatment conditions (**Figure 2E**).

Subsequently, we investigated immune infiltration by detecting CFSE+ cells and identified late-stage apoptosis using TUNEL assay. In the MSI CRC responder group, we observed a correlation between the spatial localization of a high number of infiltrating CFSE+ PBMCs (green) and TUNEL-labeled cancer cell death (white), indicating immune-mediated killing in microtumors-PBMCs co-cultured treated with anti-PD-1 (**Figure 2F**). This correlation was not observed in the three control groups. Consistently, we did not observe significant CFSE+ or TUNEL signal in the non-responder MSS CRC case. To verify the specificity of TUNEL labeling and CFSE+ signals, we co-stained for nuclei and the actin cytoskeletal network, ensuring the identification of cells and confirming intact tissue and cellular morphology respectively (**Figure 2F**).

### 3.3 *POLE* A456P mutation potentiates high tumor mutation burden and *ex vivo* response to pembrolizumab

Given recent clinical case studies reporting partial or complete responses in *POLE*-mutated MSS CRC, we aimed to investigate evidence of *ex vivo* response within our model. We received a case from a 37-year-old Caucasian male diagnosed with treatment-naïve, moderately differentiated MSS CRC harboring *POLE* mutations, who underwent surgery for tumor removal. The tumor specimen was collected, promptly sent to pathology, and then delivered directly to our laboratory for experiment. In this report, the case is labeled as MOD3, and its clinical data are presented in **Figure 3A** and **Supplementary Table S1**. NextGen sequencing data retrieved from the UF Shands Clinical and Translational Science Institute (CTSI) RedCap database (PID 11877, project title: MOD2EL v2) revealed multiple *POLE* mutations for this case. We also conducted whole exome sequencing (WES) and confirmed the presence of six missense mutations on the *POLE* full-length sequence as illustrated in **Figure 3B**. Notably, the amino acid mutation p.Ala456Pro (p.A456P; c.1366G>C) identified on exon 14, is situated in the exonuclease domain and has been suggested as a recurrent somatic aberration and identified as a pathogenic *POLE* hotspot mutation for endometrial cancer (37, 38). In addition, mutations in the *POLE* exonuclease domain are often found in MSS tumors, leading to hypermutation and associated response to immunotherapy (12). The remaining five mutations, distributed across other domains of the *POLE* gene, are of unknown significance.

In subsequent studies, we further characterized and compared the mutation profiles of additional CRC cases, including three MSS CRC MOD 4, 5 and 6. In addition, we performed an *ex vivo* experiment on an MSI CRC case namely MOD7, which also harbors *POLE* mutations, for comparison with MSS CRC MOD3. WES analysis of MOD7 unveiled the presence of two missense mutations and one splice site mutation within the *POLE* gene. The pathogenic significance of these mutations has not been reported. We presented the clinical data for these CRC cases in **Figure 3A** and **Supplementary Table S1**, along with their somatic mutation profiles in key CRC-associated genes (39) in **Figure 3C**. Although MOD3 and MOD7 share multi-hit *POLE* mutations, they demonstrate distinct driver gene profiles. Notably, MSS CRC MOD3 presents a significantly higher number of these mutations compared to other MSS CRC cases and even to MSI MOD7. MSS MOD4 and 5 exhibited the most similar driver gene mutations (TP53 and APC) and copy number alteration (CNA) profiles, with these two genes displaying the most widespread CNAs across the genome. Additionally, typical CNAs observed in CRC, such as gains in chromosome 13 (chr 13) and losses in chromosome 18 (chr 18), are evident in these samples. On the other hand, MOD6 exhibits a unique driver gene mutation profile, featuring alterations in PIK3CA, PTEN, and KRAS, along with specific CNAs, including losses in chr 1p, 17, and 18.

Furthermore, MSI CRC MOD7 with *POLE* mutations, demonstrates hypermutation with a high mutation load of 51 mutations per megabase (mutations/Mb) (**Figure 3D**). MOD7 exhibits a distinctive driver mutation profile, featuring numerous CNAs (gains in chr 1q, 2, 13), and a higher frequency of indel mutations (12 mutations/Mb), which is commonly observed in MSI CRCs. This mutation profile sets it apart from all other MSS cases in this study. Remarkably, MSS CRC MOD3 exhibits an ultra-mutated phenotype, revealing an even higher TMB with an estimated total mutation count of 11,329 and a mutation load of 236 mutations/Mb (**Figure 3D**). This is approximately more than four times that of MOD7 and approximately 70 times more than MOD4, 5, and 6 cases.

We proceeded to investigate the evidence of response to anti-PD-1 therapy for MSS CRC MOD3 and MSI CRC MOD7 using our *ex vivo* model, as described above. The workflow is illustrated in **Figure 1**, where patient whole blood and tumor tissue specimens were collected for the experiment following selection for pathology examination. IF assays at specific time points revealed microtumors with robust cell viability and intact boundaries, indicative of a well-organized and thriving culture (**Figure 3E**).

We established four conditions for the studies: (1) co-culture of MOD3 and MOD7 microtumors and patient-matched CFSE-labeled PBMCs with pembrolizumab (20 µg/mL) through continuous, unidirectional vertical perfusion. As a control (2) we also treated the co-culture with human IgG4 isotype control (ISO). In addition, (3) we treated microtumors alone with pembrolizumab (defined as: microtumors + anti-PD-1) to investigate potential anti-tumor responses stemming from TILs populations and (4) microtumors alone with ISO (defined as: microtumors + ISO ctrl) as an additional control. In line with the previous co-culture experiments, we evaluated daily perfused media for IFNγ levels, serving as an indicator of an immune-mediated anti-tumor response. Although not statistically significant, a higher trend in IFNγ production was observed in the microtumor-alone condition following anti-PD-1 treatment, suggesting a potential anti-tumor response from the TILs population (**Figure 3F**). Further investigation is needed to confirm this finding and clarify whether local immune reinvigoration contributes to effective recruitment of PBMCs and overall anti-tumor response. **Figure 3G** presents confocal IF images of a representative MOD3 microtumor cultured for 96 hours, revealing abundant CD45+ TILs in the microtumors. On the other hand, the co-culture of microtumors with PBMCs at the same anti-PD-1 dose resulted in a minimal response, possibly due to the presence of myeloid-derived suppressor cells within the PBMCs (40, 41), potentially dampening the anti-tumor effect (42, 43).

### 3.4 Intra- and inter-tumor heterogeneities

We closely examined the tumor microenvironment (TME) in the selected CRC cases to explore intra- and inter-tumor heterogeneity influencing the response to anti-PD-1 treatment. We employed multiplexed ion beam imaging (MIBI) platform to capture ten regions of interest (800 x 800µm) within formalin-fixed paraffin-embedded (FFPE) specimen sections (**Figure 4A**), ensuring a precise representation of the TME.

IHC staining of the entire tissue displayed a heterogeneous distribution of CD8+ and CD4+ T cells across all samples, with notably significant expression observed in MSI CRC MOD1 (**Figure 4B**). In addition, TILs are notably present in MSS CRC MOD3 compared to MOD2 and even MSI CRC MOD7. We applied a comprehensive 25-marker panel to conduct a thorough profiling of various immune subsets present within the complex landscape of the TME in these cases. The analyses were done blinded to patient outcome and CRC phenotype. Macroscopic region analysis initially revealed that MOD7 exhibits the highest stromal distribution, whereas MOD3 has the greatest tumor area, as quantified in **Figure 4C** and visually depicted in **Figure 4A**.

Cell segmentation and phenotyping revealed cellular heterogeneity, as evidenced by variations in cell population densities (**Figure 4D**), which shows higher cancer cell populations for MSS MOD3 and significantly high TILs in MSI MOD1. Further analysis showed significantly higher CD8+ T cell densities in MSI MOD1 compared to other samples (**Figure 4E**). Consistent with other studies, there is more PD-1 expression on CD8+ T cells for both MSI cases, MOD1 and MOD7. Remarkably, although the count of CD8+ T cells is not markedly elevated in MSS MOD3, spatial analysis of T cell distribution reveals that, on average, these immune cells are localized closer to tumor cells in both MOD1 and MOD3 cases compared to the rest of the samples (**Figure 4F**). However, these findings are limited by sample size and the number of *ex vivo* cases. Further investigations are necessary to draw a better definitive conclusion. This close cancer-immune proximity could contribute to the observed anti-tumor response to anti-PD-1 treatment in both cases. Intra- and inter-tumor heterogeneity in cell populations and phenotypes across all cases are effectively summarized and depicted in the cell density heatmap (**Figure 4G**). Notably, there is a significant elevation in the T cell population for MOD1, contrasting with MOD7 despite both sharing the same MSI CRC phenotype.

## 4 Discussion

To our knowledge, this study pioneered the development of the first *ex vivo* model involving long-term co-culture of patient-derived CRC microtumors with autologous PBMCs for screening responses to immunotherapy. The LLS microgels act as an ECM scaffold, stably upholding microtumors and immune cells in 3D, while the interstitial space between microgel particles enables unconstrained cellular activities (24, 28). In a target-specific CAR T cell killing assay, IFNγ typically peaks within the initial 24–48 hours of co-culture (22, 44, 45). However, in this study, we observed the peak IFNγ production in our system at 72 hours. This “delay” in peak IFNγ suggested a period of recovery from *ex vivo* culture for T cell activation and engagement in anti-tumor activities in response to anti-PD-1 treatment. In addition, LLS microgels are non-degradable, and T cells must navigate the tortuous microchannel network of the interstitial space between the microgels to infiltrate the microtumors via chemotaxis (28). Therefore, this platform imposes physical barriers to T cell migration in 3D, mimicking immunosuppressive properties of a TME in solid tumors (28), thereby further delaying immune-mediated antitumor function.

Response to PD-1 blockade from CRC patients diagnosed with *POLE* mutations has been reported in clinical case studies even for non-responder MSS phenotype. In most of these cases, immunotherapy was not given as a first-line treatment. Instead, the patients had to undergo other cancer treatment modalities, including neo-or adjuvant chemotherapy, which are costly and often lead to low quality of life. The application of *ex vivo* models may facilitate the rapid screening of effective drug combinations and the study of their mechanisms of action or resistance. This study presents the first evidence of an *ex vivo* response to checkpoint therapy in MSS CRC, characterized by the presence of the *POLE* A456P mutation and an additional five somatic *POLE* mutations of unknown significance. While *POLE* A456P has been suggested to be pathogenic for endometrial cancer (46), its pathogenicity remains of unknown significance for CRC (38, 47). This case in our studies exhibited significantly high TMB, four times that of MSI MOD7 and approximately 70 times more than other MSS MOD4, 5, and 6. In fact, the MOD3 case exhibits an even higher mutation load at 236 mutations/Mb compared to all existing clinical case studies of *POLE*-mutated MSS CRCs that have shown partial or complete responses to pembrolizumab (**Table 1**). The highest mutation load reported in these previous studies was 200 mutations/Mb. In addition, high TMB has been proposed as an independent predictive biomarker for the response to immune checkpoint inhibitors (3, 4, 7). Notably, the CCTG CO.26 study demonstrated a correlation between TMB and the efficacy of dual immunotherapy, suggesting potential benefits for patients with TMB > 28 mutations/Mb (5). However, questions persist regarding the underlying mechanisms of response and acquired resistance. It remains to be elucidated whether the response is primarily driven by mutation load, leading to the generation of neoantigens and subsequent immune activation.

Furthermore, an important question centers on whether PD-1 blockade will primarily boost the existing intratumoral immune cells (22, 48), or recruit new T-cell infiltration (clonal replacement) (49–51). Using paired single-cell RNA and T-cell receptor sequencing, Yost and colleagues demonstrated that the T-cell response to checkpoint inhibitors primarily depends on T-cell infiltration, rather than the reinvigoration of pre-existing tumor-specific T cells, which may be less responsive than previously thought (49, 52, 53). Building on this approach, a recent study provided extensive profiling of T-cell clonotypes across different tumor-associated tissue regions, revealing the anatomical distribution of tumor-specific T cells in patients undergoing immune checkpoint inhibitor therapy (54). These findings underscore the importance of systemic immunity, not just local immune responses, in effective T-cell recruitment and overall cancer immunotherapy (55). However, the mechanisms underlying tissue-specific immune recruitment remain poorly understood. One hypothesis is that local immune reinvigoration may precede T-cell recruitment from peripheral immune reservoirs or adjacent tissues (56). Nevertheless, the extent to which local activation is required for efficient recruitment and a robust systemic anti-tumor response remains an open question. Interestingly, although not statistically significant, we observed a notable increase in IFNγ levels in microtumors treated with anti-PD-1 in the case of MSS CRC MOD3 harboring *POLE* mutations. This finding suggests a potential anti-tumor role for the intratumoral immune cell population, aligning with observations from a recent study (22). However, this level was trivial when microtumors were co-cultured with PBMCs in the presence of the drug. This is perhaps due to immunosuppression from myeloid-derived suppressor cells (MDSCs) which are present in PBMCs (57, 58). Further investigation into MDSCs and other immunosuppressive populations in responder versus non-responder phenotypes through comprehensive profiling may provide deeper insights into the mechanisms underlying the response to anti-PD-1 therapy. On the contrary, IFNγ level was the highest in microtumor-PBMC co-culture with anti-PD-1 treatment for MSI CRC MOD1, which is enriched in TILs (**Figure 4**). While TIL enrichment is widely reported for MSI CRC, this finding further suggests potential immune reinvigoration, possibly facilitating initial immune recruitment and clonal replacement. Future studies are essential to confirm whether anti-PD-1 induces clonal replacement of effector T cells in responder CRCs.

It is feasible that the synergy between mutation load and neoantigens primes the tumor for a response to anti-PD-1. Even in small quantities, existing TILs could initiate the initial response, triggering the release of inflammatory cytokines and subsequent recruitment of more immune cells to the tumor mass. Investigation of the immune cell phenotype in each tumor, correlated with functional data from *ex vivo* treatment with immune cell therapy will improve our understanding of the mechanism(s) that govern the efficacy of immunotherapy. Particularly, it would be highly advantageous to explore the correlation between MDSC levels in both circulating and tumor-infiltrating environments in MSS versus MSI phenotypes. In addition, investigating the potential impact of *POLE* pathogenic mutations on this cell population as markers of response to immunotherapy holds substantial promise. Furthermore, combination therapy involving immune checkpoint inhibitors for MSS CRC is currently under intensive investigation (59). The *ex vivo* model may also be used to screen the efficacy of mono or combination therapy in other cancer types with autologous immune cells. Given the rarity of pathogenic *POLE* mutations in CRC, our model aids in establishing preliminary evidence of any potential response to immunotherapy.

This study has limitations, including the need for a larger sample size to improve statistical analysis and additional cases to enhance predictive power. Nevertheless, it stands out as a technically advanced investigation due to the use of a 3D media-perfused *ex vivo* platform that supports interstitial migration of immune cells (28). To our knowledge, this is the first *ex vivo* model enabling long-term 3D co-culture of patient-derived microtumors and matched PBMCs under media perfusion. In addition, while anti-PD-1 therapy is typically used for advanced metastatic MSI CRC, the treatment-naïve status of all cases in this study revealed evidence of immune activation is notable in MSI MOD1, highlighting a differential response compared to the non-responder MSI MOD7 despite sharing the same MMR status. The observed presence of intratumoral immune cells via multiplex IF and the spatial distribution of lymphocytes in *POLE*-mutated CRC further underscore the platform’s potential for mechanistic insights. These findings collectively highlight the promise of this platform for future studies aimed at unraveling tumor-immune dynamics and therapeutic responses.

## 5 Conclusion

The presence of inter- and intra-tumor heterogeneity has a profound impact on the landscape of cancer treatment options. Unfortunately, many CRC patients do not typically benefit from immunotherapy, which often leads to alternative chemotherapies and other suboptimal treatments. However, emerging evidence indicates that identifying CRC patients with pathological *POLE* mutations, exhibiting high TMB may serve as potential predictors of favorable response to immunotherapy. Further evaluation of these mutations as potential biomarkers for overall TMB, their correlation with the response to immune checkpoint blockade, and elucidation of the mechanisms of response provide additional opportunities for future research. *Ex vivo* model systems may play an important role in developing personalized cancer treatment strategies, a key factor for achieving optimal outcomes.

## Data Availability

The datasets generated in this study are included in this published article and its supplementary information files. Additional raw data are available from the corresponding author (duy.nguyen@moffitt.org) upon reasonable request. Due to patient privacy concerns, the UF Shands Clinical and Translational Science Institute (CTSI) RedCap database are not publicly available.
