# Supplementary material for: Patient-derived colorectal microtumors predict response to anti-PD-1 therapy
Source: Front Immunol. 2025 Aug 12;16:1640500. doi: 10.3389/fimmu.2025.1640500 (PMC12378724; doi:10.3389/fimmu.2025.1640500)
Supplement: Supplementary file 1 [file DataSheet1.pdf]

supplementary materials

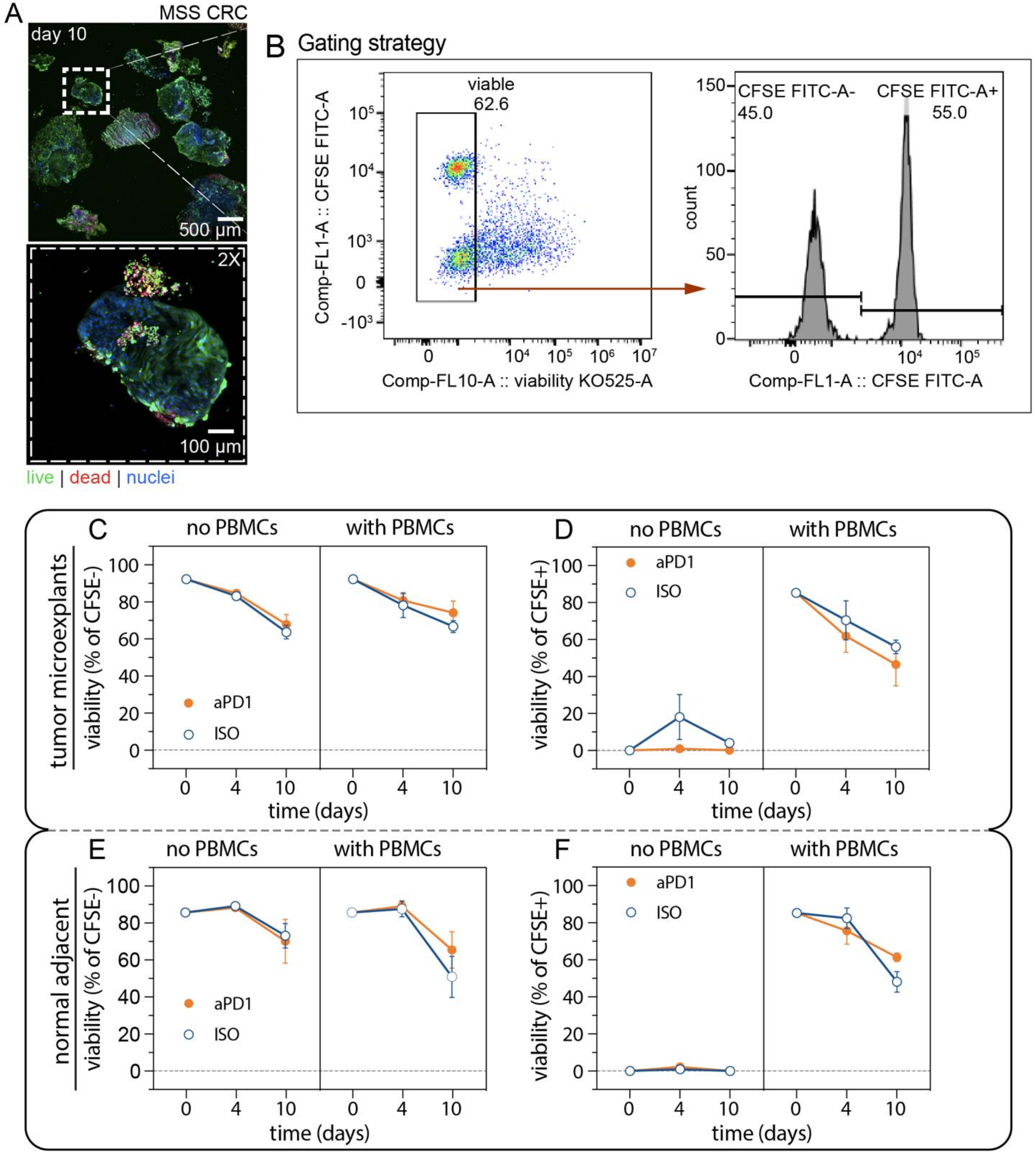

Supplementary Figure S1. Ex-vivo co-culture of patient-derived microtumors and PBMCs in 3D. A) Confocal microscopy images show the viability of representative microtumors assessed by Calcein AM (green: live), BOBO-3 Iodide (red: dead), and Hoechst 33342 (blue: nuclei). The samples were cultured in a 3D perfusion platform for 10 days before data collection. B) Flow cytometry gating strategy. C-D) Flow cytometry analyses on the viability of microtumors and PBMCs. C) Quantification of viability for CFSE- cell populations. Microtumors were cultured alone (no PBMCs) and co-cultured with PBMCs in the presence of an anti-PD-1 immune checkpoint inhibitor and isotope (IgG4) control (ISO). D) Flow cytometry analysis of the viability of CFSE+ PBMCs co-cultured with microtumors with and without anti-PD-1. E-F) Flow cytometry analyses on the viability of normal adjacent tissues and PBMCs. E) Quantification of the viability of CFSE- cells. F) Quantification of the viability of CFSE labeled PBMCs (CFSE+) co-cultured with normal adjacent tissue explants. The co-culture samples were treated with anti-PD-1 and ISO control. Note: all 'No PBMCs' samples indicate microtissues were cultured alone as controls. The microtumors were confirmed MSS CRC. Technical triplicates n=3 for all conditions unless indicated otherwise.

Supplementary Table S1. REDCap database summary for patients MOD1, MOD2, MOD3, and MOD7, including post-operative treatment history. Note: Variants of likely or unknown significance are not comprehensively listed. Full genomic and clinical reports are available in the REDCap electronic database hosted by the University of Florida. MOD: Microtissues for Oncology and Drug Discovery.

patient ID	record ID	grade	MMR status		mutation by NGS	postop-treatment
MOD1	5	2	deficient	MLH1;PMS2	BRAF c.1799T>A p.V600E VAF: 39% chr7:g.140453136A>T (Tier 1A) (pathogenic)<br>TP53 c.1146delA p.K382fs*? VAF: 42% chr17:g.7572963delT (Tier 2C) (pathogenic)<br>TP53 c.267delC p.S90fs*33 VAF: 39% chr17:g.7579420delG (Tier 2C) (pathogenic)	No adjuvant treatment recommended
MOD2	9	2	intact	N/A	APC c.4188delT p.F1396fs*19 VAF: 8.53% chr5:g.112175476delT (Tier 2D) (pathogenic)<br>APC c.847C>T p.R283* VAF: 18% chr5:g.112151204C>T (Tier 2D) (pathogenic)	XELOX; Xeloda and Oxaliplatin
MOD3	21	2	intact	N/A	APC c.4630G>T p.E1544* VAF: 18% chr5:g.112175921G>T (Tier 2C) (pathogenic)<br>APC c.4339C>T p.Q1447* VAF: 18% chr5:g.112175630C>T (Tier 2C) (pathogenic)<br>APC c.2828C>A p.S943* VAF: 18% chr5:g.112174119C>A (Tier 2C) (pathogenic)<br>EP300 c.1738C>T p.R580* VAF: 18% chr22:g.41533772C>T (Tier 2C) (pathogenic)<br>NF1 c.1783G>T p.E595* VAF: 8.62% chr17:g.29550523G>T (Tier 2C) (pathogenic)<br>NF1 c.1570G>T p.E524* VAF: 19% chr17:g.29546065G>T (Tier 2C) (pathogenic)<br>KRAS c.436G>A p.A146T VAF: 8.14% chr12:g.25378562C>T (Tier 1A) (pathogenic)<br>ATM c.5623C>T p.R1875* VAF: 16% chr11:g.108175528C>T (Tier 2C) (pathogenic)<br>PTEN c.895G>T p.E299* VAF: 18% chr10:g.89720744G>T (Tier 2C) (pathogenic)<br>PIK3R1 c.1042C>T p.R348* VAF: 18% chr5:g.67588951C>T (Tier 2C) (pathogenic)<br>POLE c.1366G>C p.A456P (likely)<br>NF1 c.7072G>T p.G2358* (likely)<br>NF1 c.6641G>T p.R2214I (likely)<br>PALB2 c.3241G>T p.E1081* (likely)<br>KMT2A c.748G>T p.E250* (likely)<br>ATM c.5480T>G p.L1827* (likely)<br>FGFR2 c.2276G>A p.R759Q (likely)<br>SMC3 c.295C>T p.R99* (likely)<br>TSC1 c.524T>C p.V175A (likely)<br>PIK3R1 c.1156C>T p.R386* (likely)<br>FAT1 c.8050G>T p.E2684* (likely)<br>FGFR3 c.1535A>G p.D512G (likely)<br>BAP1 c.176G>A p.R59Q (likely)<br>SETD2 c.2890G>T p.E964* (likely)<br>BRINP3 c.913C>T p.R305*(likely)	FOLFOX
MOD7	26	2	deficient	MLH1;PMS2	NF1 c.233delA p.N78fs*7 VAF: 17% chr17:g.29486050delA (Tier 2C) (pathogenic)<br>TP53 c.1146delA p.K382fs*? VAF: 8.31% chr17:g.7572963delT (Tier 2C) (pathogenic)<br>BRAF c.1799T>A p.V600E VAF: 14% chr7:g.140453136A>T (Tier 1A) (pathogenic)<br>ETV6 c.391dupT p.S131fs*chr12:g.12006417_12006418insT (likely)<br>SMARCA2 c.4300delA p.I1434* chr9:g.2181665delA (likely)<br>ARID1A c.2272delC p.Q758fs*chr1:g.27088659delC (likely)	No adjuvant treatment recommended
